# Papillary fibroelastoma arising from the coumadin ridge

**DOI:** 10.15171/jcvtr.2017.20

**Published:** 2017-04-12

**Authors:** Mahim Malik, Konstantin Shilo, Ahmet Kilic

**Affiliations:** ^1^Division of Thoracic Surgery, Department of Surgery, The Ohio State University Wexner Medical Center, Columbus, OH, USA; ^2^Department of Pathology, The Ohio State University Wexner Medical Center, Columbus, OH, USA

**Keywords:** Fibroelastoma, Tumor, Left Atrium

## Abstract

Cardiac papillary fibroelastomas (CPF) are rare cardiac tumors, mostly found on the valvular surfaces in the heart. These tumors are frond like in nature and are benign, intracardiac masses, rarely causing any hemodynamic disturbances. However, excision of these masses is indicated due to their propensity to embolize. We present a case report of the tumor found on the coumadin ridge, causing transient ischemic attacks in a patient. We performed complete excision of the tumor via median sternotomy on cardiopulmonary bypass support with cardiac arrest. The diagnosis was confirmed by histological examination. The patient had an uneventful postoperative course and was discharghed on postoperative day 4. She has had complete resolution of her symptoms post excision. The diagnosis of the mass was confirmed on histological examination.

## Introduction

Cardiac papillary fibroelastomas (CPF) are rare tumors that usually arise on a valve leaflet and less commonly on the ventricular septum or the aortic wall.^1^ Even though they are considered benign, resection of these tumors is recommended because of their propensity to embolize vasculature of vital organs.^2^

Herein we present a case of a 71-year-old female with symptoms of transient ischemic attacks. She was found to have a CPF arising from the so-called coumadin ridge (between the left atrial appendage and left superior pulmonary vein) that underwent surgical resection.

## Case Report

**Figure 1:**
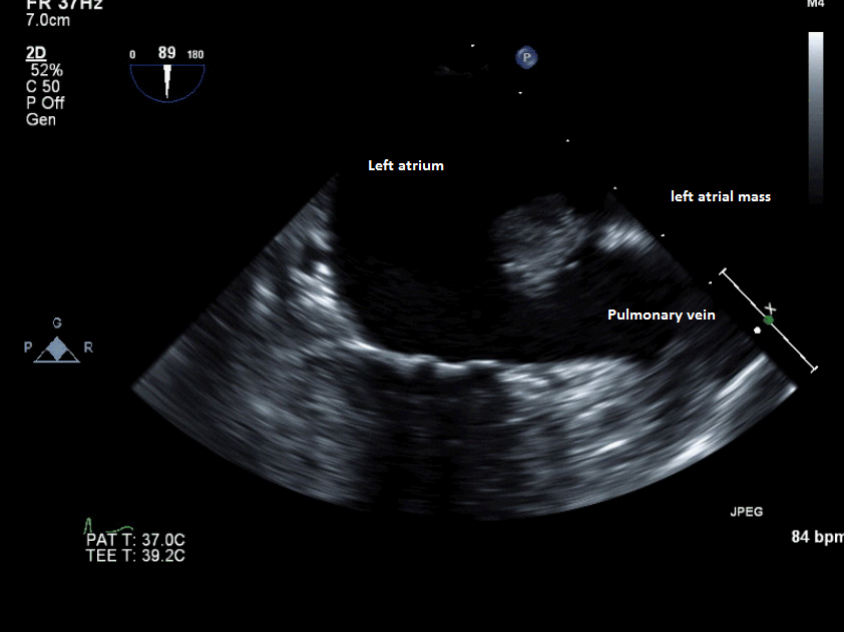
TEE image showing mobile mass in the left atrium.

**Figure 2:**
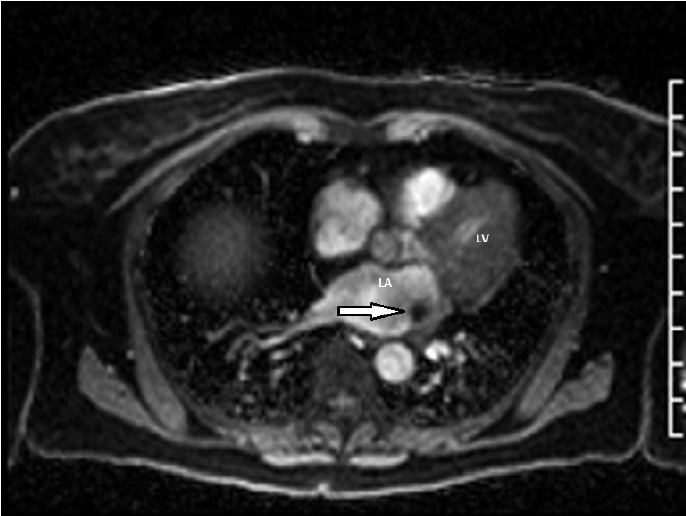
MRI imaging showing mass in the left atrium (arrow pointing toward mass).

A 71-year-old female presented to her primary care physician with complaints of lightheadedness. She had no other symptoms and her physical examination was unremarkable. A non-contrast head computed tomography scan was negative for any abnormalities. This prompted a cardiac work up for her symptoms. An electrocardiogram revealed premature ventricular contractions that led to a transthoracic echocardiogram (TTE). This showed a 1 cm mass within the left atrium, concerning for a thrombus. A transesophageal echocardiogram (Figure 1) with subsequent cardiac magnetic resonance imaging, was performed to better characterize the mass. This revealed a mass attached to the ridge between the left atrial appendage and superior pulmonary vein by a discrete stalk, measuring 1.4 x 1.1 x 1.0 cm. The mass appeared hypovascular on perfusion imaging, with no specific enhancement with delayed enhancement imaging (Figure 2). Her preoperative workup was completed with a left heart catheterization that did showed nonobstructive coronary anatomy. She was scheduled for elective resection of this mass.

**Figure 3:**
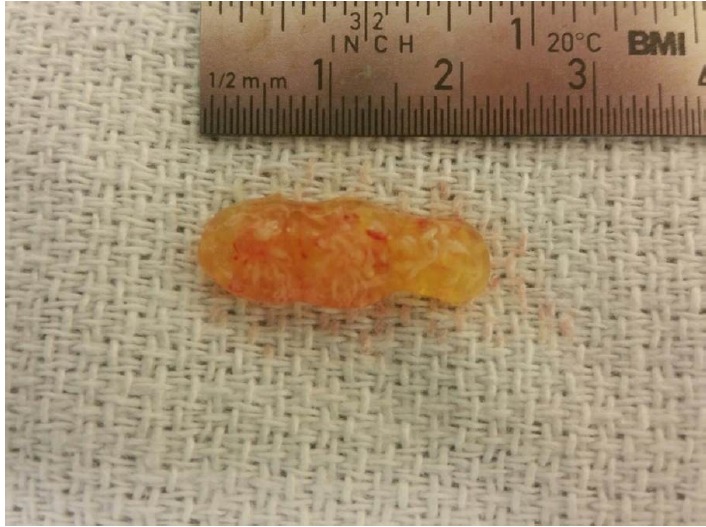
Gross appearance of tumor after resection.

The procedure was performed via median sternotomy with the aid of cardiopulmonary bypass. Central aortic arterial cannulation with bicaval venous cannulation was performed. The heart was arrested with aid of antegrade cardioplegia. For surgical exposure, Sondergaard’s groove was dissected and a left atriotomy performed. A 1 cm mobile, gelatinous mass on the left atrial wall was found (Figure 3). This arose between the left atrial appendage and the left superior pulmonary vein. The lesion was resected en bloc, along with the stalk and a margin of the left atrial wall. The defect was closed using a 4-0 monofilament suture. The immediate post-operative course was uneventful and the patient was discharged home on post-operative day 4. On outpatient follow up she has been doing well and has remained asymptomatic. A repeat echocardiogram is scheduled to be performed at one year follow up.

**Figure 4:**
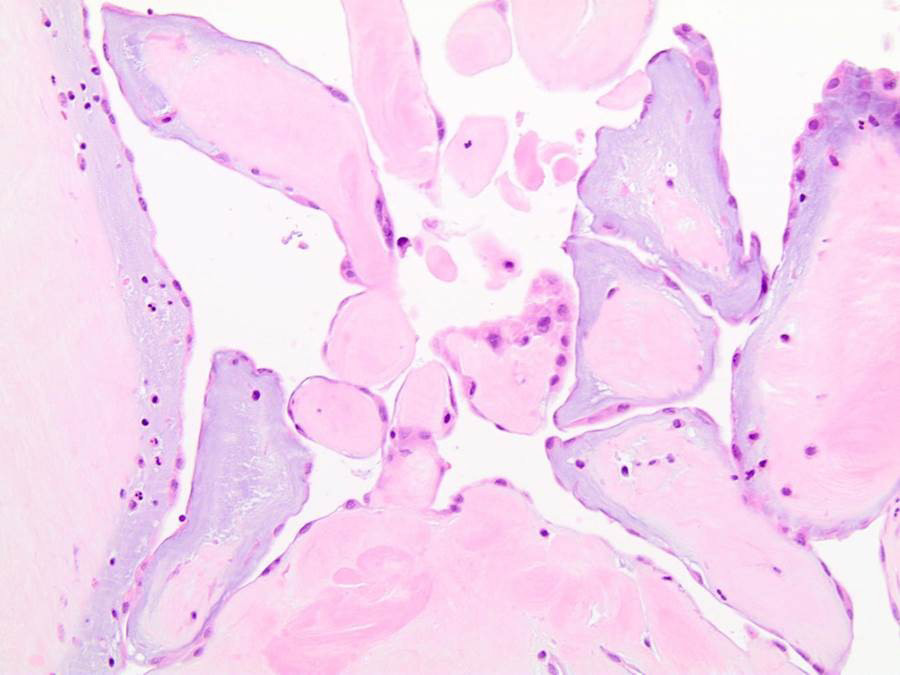
Histological features of papillary fibroelastoma.

On gross examination, the tumor was soft and grey-white, that appeared frond-like and translucent when submerged in liquid. Histologically, it was composed of branching avascular papillae with variable amount collagen and myxoid change consistent with papillary fibroelastoma, with no evidence of extension into the left atrial wall (Figure 4).

## Discussion

CPF are rare tumors that are usually asymptomatic and often discovered incidentally. A large retrospective analysis of 725 patients revealed the valvular surfaces as the most common location of these tumors, with only ten arising from the left atrial wall.^3^ Since greater than 95% of CPF are located on the left side, these usually present with symptoms of systemic embolism, with reports of tumors fragments embolizing to the cerebral, coronary and even mesenteric circulation.^3^ Nevertheless, there have been reports of right sided tumors embolizing to the pulmonary circulation.^4^ It has been suggested that that the tumor location may influence the potential for thromboembolic events, in particular in the vicinity of the left atrial appendage. Thrombus formation and subsequent embolization may be more susceptible due to low flow in the area of the left atrial appendage.^2^

Although CPF are the second most common benign tumors of cardiac origin, the left atrium is a very unusual location for these tumors.^3^ A survey of recent literature revealed less than 20 reports of CPF arising from the left atrium.^5-8^ The presenting symptoms were transient ischemic attacks in most cases. Rarely, these tumors may present with chest pain, angina or even myocardial infarction, if embolism to the coronary arteries occurs.^7^

Diagnosis is usually made by echocardiography, either transthoracic or transesophageal, with images confirming a pedunculated and mobile mass. This may appear speckled, with a stippled pattern near the edges.^1^ More recently, cardiac magnetic resonance imaging has helped differentiate these tumors from other cardiac masses.^9^ Electrocardiographic findings are usually non specific, but may present with atrial arrhythmias. Due to the risk of embolism with these tumors, surgical excision is the recommend therapy.

The coumadin ridge has been described as a ridge of atrial tissue separating the left atrial appendage from the left pulmonary vein. It can appear as a mass that protrudes into the left atrium and can appear similar to a tumor or thrombus.^6^ This structure has often been mistaken as a thrombus, resulting in life long anticoagulation for the patient. In these situations, contrast magnetic resonance imaging is helpful, and aids in diagnosis.^5^

In conclusion, left atrial papillary fibroelastomas are rare benign cardiac tumors, with propensity to cause thromboembolic events. In patients with unexplained neurological symptoms, echocardiographic imaging should be performed. Surgical excision is warranted in these cases since these small, seemingly harmless tumors have the potential to cause significant systemic and neurologic complications.

## Ethical approval

This study is in compliance with the Ohio State University Internal Review Board Committee Rules and Regulations.

## Competing interests

None.

## References

[1] Kouchoukos NT, Blackstone EH, Hanley FL, Kirklin JK. Kirklin/Barratt-Boyes Cardiac Surgery. Saunders; 2012.

[2] Mohammadi S, Martineau A, Voisine P, Dagenais F (2007). Left atrial papillary fibroelastoma: a rare cause of multiple cerebral emboli. Ann Thorac Surg.

[3] Gowda MR, Khan IA, Nair CK, Mehta NJ, Vasavada BC, Sacchi TJ (2003). Cardiac papillary fibroelastoma: a comprehensive analysis of 725 cases. Am Heart J.

[4] Hakim FA, Aryal MR, Pandit A, Pandit AA, Alegria JR, Kendall CB (2014). Papillary fibroelastoma of the pulmonary valve - a systemic review. Echocardiography.

[5] McKay T, Thomas L (2008). ‘Coumadin ridge’ in the left atrium demonstrated on three dimensional transthoracic echocardiography. Eur J Echocardiogr.

[6] Waziri F, Grove EL (2014). Left atrial papillary fibroelastoma as an unusual cause of myocardial infarction. BMJ Case Rep.

[7] Saitoh Y, Soeda T, Setozaki S, Harada H, Miura H (2013). Left atrial papillary fibroelastoma accidentally diagnosed with gastric cancer. Ann Thorac Cardiovasc Surg.

[8] Strecker T, Rösch J, Weyand M, Agaimy A (2012). Primary and metastatic cardiac tumors: imaging characteristics, surgical treatment, and histopathological spectrum: a 10-year-experience at a German heart center. Cardiovasc Pathol.

[9] Kamina T, Takeshita T, Kimura I (2003 Dec). Role of magnetic resonance imaging for evaluation of tumors in the cardiac region. Eur Radiol.

